# Case management intervention of high users of the emergency department of a Portuguese hospital: a before-after design analysis

**DOI:** 10.1186/s12873-022-00716-3

**Published:** 2022-09-13

**Authors:** Simão Gonçalves, Francisco von Hafe, Flávio Martins, Carla Menino, Maria José Guimarães, Andreia Mesquita, Susana Sampaio, Ana Rita Londral

**Affiliations:** 1 Value for Health CoLAB, NOVA Medical School, Lisbon, Portugal; 2 Nova School of Science and Technology, Nova University of Lisbon, Lisbon, Portugal; 3 CHRC, Comprehensive Health Research Centre, NOVA Medical School, UNL, 1099-085 Lisbon, Portugal; 4 Nova LINCS, Nova School of Science and Technology, Universidade Nova de Lisboa, Lisbon, Portugal; 5 Hospital Garcia de Orta, EPE, Almada, Portugal; 6 Unidade de Saúde Familiar Cova da Piedade, Almada, Portugal

**Keywords:** Case management; Integrated care; High users; Emergency department; Costs; Healthcare system sustainability

## Abstract

**Background:**

Emergency department (ED) High users (HU), defined as having more than ten visits to the ED per year, are a small group of patients that use a significant proportion of ED resources. The High Users Resolution Group (GRHU) identifies and provides care to HU to improve their health conditions and reduce the frequency of ED visits by delivering patient-centered case management integrated care. The main objective of this study was to measure the impact of the GRHU intervention in reducing ED visits, outpatient appointments, and hospitalizations. As secondary objectives, we aimed to compare the GRHU intervention costs against its potential savings or additional costs. Finally, we intend to study the impact of this intervention across different groups of patients.

**Methods:**

We studied the changes triggered by the GRHU program in a retrospective, non-controlled before-after analysis of patients’ hospital utilization data on 6 and 12-month windows from the first appointment.

**Results:**

A total of 238 ED HU were intervened. A sample of 152 and 88 patients was analyzed during the 6 and 12-month window, respectively. On the 12-month window, GRHU intervention was associated with a statistically significant reduction of 51% in ED visits and hospitalizations and a non-statistically significant increase in the total number of outpatient appointments. Overall costs were reduced by 43.56%. We estimated the intervention costs to be €79,935.34. The net cost saving was €104,305.25. The program’s Return on Investment (ROI) was estimated to be €2.3.

**Conclusion:**

Patient-centered case management for ED HU seems to effectively reduce ED visits and hospitalizations, leading to better use of resources.

**Supplementary Information:**

The online version contains supplementary material available at 10.1186/s12873-022-00716-3.

## Background

Emergency Department (ED) High Users (HU) are a small group of patients that use a significant proportion of ED resources through multiple recurrent visits [1–3]. Studies estimate that HU “comprise 4.5% to 8% of all ED patients while accounting for 21% to 28% of all visits” [4]. In Portugal, 12% of patients who visited the ED in 2015 did so at least four times and were responsible for 35.9% of all ED visits [5].

HU may contribute to aggravating the already existing ED overcrowding issues [6–9], resulting in reduced quality of care, increased waiting times, and healthcare professionals’ stress [2, 10]. Consequently, ED HU may compromise access to the ED for patients with life-threatening situations whose condition could deteriorate if not treated on time [10–15]. The high burden that HU place on the healthcare system also leads to excessive hospital costs, so allocating resources to the ED could reflect a questionable stewardship of resources, as HU should be treated in other settings [11, 16, 17]. In addition, ED HU have been shown to visit other non-emergency care services more frequently than non-ED HU [18, 19].

HU often have complex healthcare needs that cannot be optimally managed in an ED that provides episodic and discontinuous care [1, 11, 15, 17, 19]. Therefore, understanding the health characteristics of these patients is necessary to improve the quality of care (in ED or in primary care). Literature reports several characteristics that correlate with ED high usage, namely: psychiatric and physical conditions, chronic diseases, advanced age, lack of family support, substance abuse, socioeconomic status, and demographic and socio-cultural characteristics [1, 2, 6, 10, 12, 14, 16, 19–24]. Furthermore, ED HU report higher mortality and worse health status and outcomes [3, 6, 19, 21, 25].

Interventions such as case management have been shown to be effective in reducing the frequency of ED HU visits [6, 26]. In addition to the patient’s clinical perspective, these interventions also focus on the socioeconomic, emotional, and environmental aspects of the visit [13]. This care strategy has shown promising results in reducing hospital use and costs, increasing patient satisfaction and quality of life, and [20, 23] positively impacting the healthcare system [26]. However, it is unknown whether care management interventions are as effective in high frequency HU patients compared to lower frequency ED patients.

## Objectives

The study's main objective was to measure the impact of the High Users Resolution Group ( GRHU) intervention on ED visits. Additionally, we intended to provide an overall analysis of the impact of the GRHU intervention on different hospital services, such as outpatient appointments and hospitalizations.

As a secondary goal, we aimed to study the impact of this intervention regarding the program costs against the potential savings or additional costs. Finally, we intended to study the impact of the intervention across patient groups to understand better which patients may have better or worse outcomes from the intervention.

## Methods

### Intervention

Portugal has a tax-funded NHS that provides coverage to all residents. Considering the growing health expenditures in Portugal [27], it is necessary to guarantee that the available resources are being optimally used, reducing resource waste, for example, by reducing unnecessary ED visits.

In 2016, Hospital Garcia de Orta (HGO) and the Agrupamento de Centros de Saúde Almada-Seixal (ACES—Almada-Seixal) created a program to provide case management interventions to HGO’s HU. The High Users Resolution Group Program (GRHU) is a multidisciplinary team that identifies and provides care to HU aiming to improve their health status and, consequently, reduce their visits to the ED. GRHU addresses HU's healthcare and social needs by delivering patient-centered case management interventions [28]. The program’s team comprises four social workers, six doctors (three general physicians, two internists, one psychiatrist) and four nurses. Their workflow includes: i) discussing potential patients to include in the program; ii) discussing and planning personalized steps to tackle the needs of each HU included in the program (Integrated Case Plan (ICP)); and iii) assigning a Case Manager (CM) to each HU to implement the ICP through outpatient consultations. CM’s role in the GRHU project is to: i) collaborate with the patient’s primary healthcare center professionals to implement the ICP; ii) collaborate with HGO’s different specialties to implement the ICP; iii) collaborate, when needed, with other healthcare providers or community services; and iv) collaborate with the patients’ informal caregivers; v) monitor the patient to avoid ED visits. GRHU has eleven CMs responsible for a maximum of eight patients each. Between June 2016 and February 2020, the GRHU team performed, on average, five appointments per month where they defined the ICP for the new patients that were included in the GRHU program.

### Study setting and population

The study was approved by the HGO’s Ethical Committee. Anonymized data from HU patients (patients with over ten ED visits in a single year at a given time) between June 2016 and February 2020 was made available for the study. This period refers to the beginning of the GRHU program and the date of data the request. Data included demographic and hospital service usage information. We performed a retrospective non-controlled before-after analysis of patients’ ED visits data on 6 and 12-month windows from the intervention. We defined the 6 or 12 months before the first appointment as the before period, while the 6 or 12 months after was the after period. These two periods of analysis were selected as the GRHU team was interested in understanding the results of their intervention in both short- and long-term settings. The study was conducted at HGO, a public hospital in Almada, Portugal, with approximately 164 thousand ED visits in 2020 [29]. Data included the 972 ED patients that were HU since the beginning of the GRHU program. The GRHU team selected 238 patients to participate in the program. To be eligible to participate in this program, patients need to be at least 18 years old, have visited the ED at least 10 times in a single year during the 4-year data collection period, and live in the HGO’s area of influence. Then, if the patient agreed to participate in the study, he signed an informed consent and was interviewed by the GRHU team. During the interview, the GRHU team tried to understand the patient’s needs and involve him in designing his ICP.

### Inclusion and exclusion criteria

For our analysis, we included all patients in the GRHU program (238 patients) that had at least one visit to the hospital during the before period. Patients who died during the after period were excluded to prevent being wrongfully considered as positive signals in the reduction of episodes. As reported in the literature there was a significant drop in ED visits in Portugal, after 29th February 2020 [30], coincident with the first COVID-19 lockdown. For this reason, patients that had data after this day were excluded to avoid any misunderstanding in interpreting ED visits’ variation.

### Data analysis

All patients that met the inclusion criteria were included in the analysis, independently of the received treatment (intent-to-treat) [31]. We performed one-tailed paired t-tests for the reduction in the mean to compare the utilization of hospital services in the before and after period. Moreover, we assessed the impact of this intervention on the clinical severity of the ED visits using the Manchester Triage System. This system prioritizes patients on five different levels: red (immediate), orange (very urgent), yellow (urgent), green (standard), and blue (non-urgent) [32].

Records of ED visits contained an ICD-9 primary diagnosis [33]. However, ICD-9 diagnoses were not registered for either most outpatient visits or hospitalizations. As such, in order to maintain uniformity, ED visits, outpatient visits and hospitalizations were grouped by the ICD-9 chapters or by clinical specialty. Collapsing individual diagnoses into their logical ICD-9 chapters also significantly reduced the number of diagnoses to a statistically manageable level.

Finally, we also analyzed the demographic characteristics of the patients to profile them: age, gender, and patient charges payment exemption. Despite being a public healthcare system, in Portugal patients must pay user-fees when they visit the ED. However, patients are exempt from paying user-fees if they report economic insufficiency, i.e., if the monthly income, divided by the number of people living in the household, does not exceed €653.64 [34], or patients that have at least 60% of physical or mental disability, declared by an independent medical committee.

We conducted regular meetings with the GRHU team to discuss the obtained results from the data analysis.

### Economic analysis

We estimated the GRHU program savings or additional costs as the difference between the costs before and after the intervention per patient, using the hospital perspective, i.e., focusing the analysis on the costs and savings incurred by the hospital, as the hospital was the financier of the GRHU program [35]. The cost categories included ED visits, hospitalizations, and outpatient appointments, their costing information was retrieved from HGO’s Long-term Contract Program (2017–2019). This contract determines the cost of each clinical procedure based on the expected cost for the hospital to treat each diagnosis [36].

Regarding the GRHU intervention costs, the hospital provided the number of hours per week devoted by each GRHU team member (including the time devoted to appointments with patients and the necessary time to prepare them) and their monthly salary. We assumed that the number of weekly hours devoted to the program was the same for every 52 weeks of the year. We computed the cost of each Human Resource (HR) per hour by dividing the number of working hours per month (assumed to be 140 h, 35 h per week) by their monthly salary. However, as it is estimated that costs with HR represent 60% of the total operating costs [37], we added 40% of other costs (that represent other direct and indirect costs) to the HR ones.

We estimated the Return on Investment (ROI) of the GRHU intervention as the ratio between the savings or costs that it generates and its cost [14]. All monetary values are in Euros as of 2020.

## Results

### Sample selection and characteristics

**Fig. 1:**
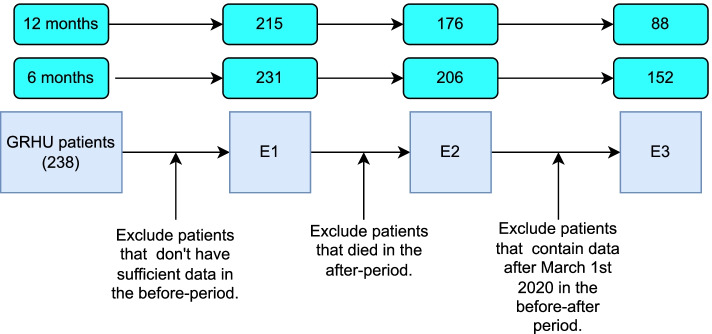
Exclusion steps for the before-after analysis

A total of 238 adult patients participated in the GRHU program between the 26th of June 2016 and the 29th of February 2020. Out of the 238 patients, after applying the exclusion criteria, we included 152 patients for the 6-month and 88 patients for the 12-month in the before-after analysis (Fig. 1).

Twenty six percent (26%) of the patients in the GRHU program died during the period of the analysis, compared to 35% of deaths in the group of patients from our database that were not in the program.

As there were no significant differences between the 6-month and the 12-month data, only the 12-month data are presented below. Results for the 6-month window can be found in Appendix 1.

Fifty eight percent of the 88 patients included in the twelve-month window analysis were male and their median age at the first visit was 58.

Most patients were in economic insufficiency (57%) (Table 1).

**Table 1:** Demographic Information of 3 groups of GRHU patients: all patients, those eligible for the six months analysis, and those eligible for the 12 months analysis

		Sample		
		GRHU patients	6 months patients	12 months patients
Age	Average	58.3	56.8	57.3
	std	18.2	17.8	17.7
Gender	Males	128	90	55
	Females	110	62	33
Payment Exemption	Economic Insufficiency	115	80	50
	Incapacity	27	17	11
	None	95	54	27

### ED utilization

We observed a statistically significant 51% reduction in the number of ED visits for the 12 month-window analysis. The median number of ED visits reduced from 14 to 7. The total number of visits per patient ranged between 8 and 45 in the before-window and between 0 and 32 after (Table 2). The most common diagnoses were: “Symptoms involving respiratory system and other chest symptoms” and “Anxiety, dissociative and somatoform disorders”.

**Table 2:** 12 months a before-after variation of 88 patients

	**Before**	**After**	**Variation**
ED episodes	1413	688	-51%*
Inpatient episodes	105	51	-51%*
Outpatient appointments	522	732	+ 40%
Outpatient appointments without GRHU	522	508	-3%

^*^ *p* < 0.05, one-tailed t-test for the reduction in mean

We further analyzed ED episodes across the Manchester triage system colors (Table 3). All colors registered a decrease in the number of ED visits. However, the decrease was only statistically significant for the yellow, green, and orange levels (58%, 52%, and 37%, respectively).

**Table 3:** 12 months before-after variation of 88 patients across Manchester triage system colors

	**Before**	**After**	**Variation**
Yellow	584	248	-58%*
Green	532	253	-52%*
Orange	208	132	-37%*
Blue	68	48	-29%*
Red	3	2	-33%

^*^ *p* < 0.05, one-tailed t-test for the reduction in mean

Furthermore, grouping patients by the primary diagnostic of each ED visit into the ICD-9 chapters reduced the analysis’ granularity from 476 unique diagnostic codes to just 19 chapters. Table 4 contains the episode reduction across the ICD-9 Chapters.

**Table 4:** Reduction across ICD-9 Chapters in the 12 months before-after analysis

	**Before**	**After**	**Variation**
Diseases of the Digestive System	54	21	-61%*
Mental Disorders	237	97	-59%*
Diseases of the Genitourinary System	121	52	-57%*
Injury and Poisoning	133	61	-54%*
Supplementary Classification of Factors Influencing Health Status and Contact with Health Services	63	29	-54%*
Symptoms, Signs, and Ill-defined Conditions	331	169	-49%*
Diseases of the Circulatory System	95	52	-45%*
Diseases of the Respiratory System	98	67	-32%*
Neoplasms	5	1	-80%
Diseases of the Blood System and Blood-forming Organs	26	6	-77%
Supplementary Classification of External Causes of Injury and Poisoning	17	7	-59%
Disease of the Skin and Subcutaneous Tissue	12	5	-58%
Diseases of the Nervous System and Sense Organs	74	33	-55%
Endocrine, Nutritional and Metabolic Disease and Immunity Disorders	34	16	-53%
Diseases of the Musculoskeletal System and Connective Tissue	100	61	-39%
Infectious and Parasitic Disease	12	11	-8%

^*^ *p* < 0.05, one-tailed t-test for the reduction in mean

### Other hospital services utilization

We observed a 51% reduction in the number of inpatient stays (Table 2), corresponding to a median reduction from 1 to 0 episodes. Inpatient stays are associated with a clinical specialty group related to the nature of the episode (psychiatry, surgery, or others). Therefore, we also analyzed the episodes’ reduction across these groups (Table 5). The reduction was statistically significant in general surgery and psychiatry (78% and 69%, respectively). The average Length of Stay (LOS) before the intervention was 13.9 days, and this number decreased to 9.4 days in the after-period, resulting in a 34% reduction in LOS.

**Table 5:** Reduction across specialty groups of inpatient stay episodes in 12 months before-after analysis

	**Before**	**After**	**Variation**
General Surgery	18	4	-78%*
Psychiatry	16	5	-69%*
Internal Medicine	41	23	-44%
Urology	4	3	-25%
Cardiology	6	6	0%
Nephrology	2	2	0%
Neurology	1	1	0%
Pneumology	4	6	+ 50%

^*^p < 0.05, one-tailed t-test for the reduction in mean

Regarding the outpatient appointments (Table 2), we conducted two analyses: all appointments (including GRHU appointments) and without including the GRHU appointments. If we do not include the GRHU outpatient appointments, we observe a 3% decrease (not statistically significant). However, if we include the number of GRHU appointments, the total number of outpatient appointments grows by 41% (Table 5), which was not statistically significant. The number of outpatient appointments in the 12-months window ranged between zero and 29 in the before-period and 0 and 36 in the after-period.

### Economic analysis

The total cost of ED visits, outpatient consultations, and inpatient stays was calculated before and after the first GRHU appointment (Table 6).

**Table 6:** Total Healthcare expenditure before and after the intervention for the 12-month window

	**Before**	**After**	**Variation**
Total	€423,004.61	€238,764.02	-€184,240.59 (-43.56%)
ED	€142,742.65	€69,045.47	-€73,697.18 (-51.63%)
Outpatient appointments	€37,964.51	€52,901.62	€14,937.11 (39.34%)
Inpatient stay	€242,297.45	€116,816.93	-€125,480.52 (-51.79%)

For the 12-month window total cost for the 88 patients was reduced by 43.56%,, generating a saving of €184,240.59. Costs decreased for ED and inpatient stays (51.63% and 51.79%, respectively) and increased for outpatient appointments (39.34%).

The total cost of the GRHU program (238 patients) was €162,847.82 (€684.23 per patient). However, the total cost for the 88 included patients was Hence, the net cost saving generated by this intervention was €104,305.25. The ROI of the GRHU program was estimated to be 184,240.59/79,935.34, €2.3. This result means that for every €1 invested in the GRHU program, the hospital saved €2.3.

## Discussion

The 51% reduction in ED episodes demonstrates that GRHU’s program was successful, leading to a statically significant reduction in ED usage and inpatient hospitalization, inpatient LOS, and hospital costs. These results are similar to other case management interventions for tackling HU [1, 38]. As ED visits influence hospital re-admissions, reducing them had a spillover effect on other hospital departments (in-patient hospitalization), benefiting other hospital users, and contributing to reducing hospital overcrowding [14, 23]. We observed an increase in outpatient appointments, which were fully explained by the GRHU appointments. This program seems cost-saving, generating a saving of €2.3 per euro spent on the 12-month window, which is in line with similar studies [14]. GRHU intervention was successful in reducing ED episodes related to mental disorders diagnoses. Furthermore, there were high discrepancies when comparing the reductions among the ICD-9 Chapters. For example, diagnoses within the”Disease of the Digestive System”” were reduced by 61%, whereas “Diseases of the Respiratory System” were reduced by 32%.However, not all episodes were categorized into ICD-9 Chapters. We observed that data collapsed into ICD-9 chapters was welcomed by the GRHU team as it provided them with information that might help them improve their CM strategy for patients whose diagnoses fell into the low reduction groups. Lastly, despite the focus on reducing ED visits, inpatient stays were also reduced at the 6 and 12-month follow-ups.

This information is crucial for understanding how performing case management interventions can provide adequate treatment to the complex needs of HU. Case management effectively reduced HU necessity of returning to the ED while reducing hospital costs and crowding. Moreover, these positive results can serve as a benchmark to justify the implementation of this program on a larger scale.

Despite the promising results obtained in this study, we recognize some limitations that influence their interpretation. The underlying assumptions and weaknesses of a before-after design are well-known [39]. We consider three threats to the validity of this study. One is the history threat, in which other influential events could have affected the outcome instead of the intervention itself. This could happen, for example, due to the seasonality of the hospital ED visits, in which the winter season usually comes with more visits. However, we mitigated the seasonality risk as the GRHU interventions were spread over three years, resulting in the before-after change being computed throughout many different periods, thus reducing the risk of seasonality and isolated events influencing the event results. Another threat to the validity of this study is the regression to the mean. One selection criteria for assigning new patients to the GRHU program consisted of choosing the higher ED users at the time according to the highest ED user at the time, in contrast to randomly selecting high user patients for the program. Therefore, our findings do not necessarily represent all HU of the hospital, but only the very high users. Therefore, this is, by definition, an outlier sample of the patients. Also, we excluded patients that died during the period and those that had data after the 29th of February 2020 in the after-window. Although both decisions were taken to remove the confounding sharp reduction of hospital utilization, either caused by patient death or by the COVID-19 lockdown, they resulted in a reduction of the original sample size, this may compromise the generalization of the results.Therefore, considering these three points, we conclude that this could potentially bias our results [4]. Moreover, the GRHU team designed the program according to the needs of HGO’s patients, and we analyzed the results according to the hospital payment scheme. Again, generalization issues may arise. Due to time and COVID-19 constraints, the hospital did not provide all relevant cost information. This led to the use of different assumptions and hypotheses. To minimize this limitation, we presented the cost analysis to the hospital administration board members to validate all the assumptions. Furthermore, costs should be analyzed from a societal perspective, assessing the impact of the intervention on other relevant stakeholders, such as other hospitals or primary health care centers. Finally, by only collecting the information on hospital usage, we cannot assess the impact of the intervention on other relevant health outcomes (clinical outcomes, quality of life). Future research should also incorporate these in the analysis. This study highlights the importance of data sharing in healthcare, strengthening the multidisciplinary work between clinicians, administrators, and researchers. Data sharing opens opportunities to conduct research to enable more sustainable and higher-quality healthcare systems.

Future research should develop tools that help hospital staff select new patients for the program. Thus, optimizing their work in two ways: choosing patients that contain mainly diagnostics that experienced a high reduction in previous patients of the program and reworking the intervention to improve the reduction in diagnostic groups that do not experience a significant reduction. To do so, it is also important to consider patients’ reasons to participate or not in this program. Moreover, a study conducted with a larger patient sample that is randomized and collects outcomes and costs from a broader perspective should be implemented.

## Conclusions

The GRHU’s program focused on creating a multidisciplinary team that aimed to reduce the number of ED visits of patients that went to the ED more than ten times in the previous year. The intervention led to a 51% reduction in the number of ED visits and inpatient. Moreover, the GRHU program generated savings of €2,3 per €1 spent.

## Supplementary Information

**Additional file 1:** **A.1** Results for the analysis conducted for the 6-month window. **Table 1.** 6 months a before-after variation of 152 patients. **Table 2.** 6 months a before-after variation of 152 patients across Manchester triage system colors. **Table 3.** Reduction across specialty grouped of inpatient stay episodes in 6 months before-after analysis. **Table 4.** Total Healthcare expenditure before and after the intervention for the six-month window **Table 5.** Reduction across ICD-9 Chapters in the six months before-after analysis for 152 patients.

## Data Availability

The data that support the findings of this study are available from Hospital Garcia de Orta, but restrictions apply to the availability of these data, which were used under license for the current study, and so are not publicly available. Data may be however available from the corresponding author upon reasonable request and with permission of Hospital Garcia de Orta.
