# Supplementary material for: Case management intervention of high users of the emergency department of a Portuguese hospital: a before-after design analysis
Source: BMC Emerg Med. 2022 Sep 13;22:159. doi: 10.1186/s12873-022-00716-3 (PMC9470068; doi:10.1186/s12873-022-00716-3)
Supplement: Supplementary file 1 — Additional file 1: A.1 Results for the analysis conducted for the 6-month window. Table 1. 6 months a before-after variation of 152 patients. Table 2. 6 months a before-after variation of 152 patients across Manchester triage system colors. Table 3. Reduction across specialty grouped of inpatient stay episodes in 6 months before-after analysis. Table 4. Total Healthcare expenditure before and after the intervention for the six-month window Table 5. Reduction across ICD-9 Chapters in the six months before-after analysis for 152 patients. [file 12873_2022_716_MOESM1_ESM.pdf]

Appendix 1

A.1 Results for the analysis conducted for the 6-month window.

Table 1 6 months a before-after variation of 152 patients.

	Before	After	Variation
ED episodes	1530	627	-59%*
Inpatient episodes	100	5146	-54%*
Outpatient appointments	427	408	-4%
Outpatient appointments w/ GRHU	427	688	+61%

Table 2 6 months a before-after variation of 152 patients across Manchester triage system colors.

	BEFORE	AFTER	Variation
Green	590	254	-57%*
Yellow	614	199	-68%*
Orange	217	115	-47%*
Blue	81	49	-40%*
White	23	9	-61%*
Red	5	1	-80%

Table 3 Reduction across specialty grouped of inpatient stay episodes in 6 months before-after analysis.

	Before	After	Variation
Gastroentherology	7	1	-86%*
Psychiatry	16	3	-81%
General Surgery	17	4	-76%*
Internal Medicine	37	17	-54%
Nephrology	3	1	-67%
Cardiology	6	5	-17%
Pneumology	3	3	0%
Urology	3	6	+100%
Neurology	1	2	+100%

Table 4 Total Healthcare expenditure before and after the intervention for the six-month window

	Before Intervention	After Intervention	Difference (%)
Total	€415,457.84	€218,167.06	-€197,290.78 (-47.49%)
ED	€154,204.05	€62,957.38	-€91,246.67 (-59.17%)
Outpatient Appointments	€30,981.34	€49,756.27	€18,774.93 (60.60%)
Inpatient stay	€230,272.45	€105,453.41	-€124,819.04 (-54.20%)

Table 5 Reduction across ICD-9 Chapters in the six months before-after analysis for 152 patients.

	Before	After	Variation
**Diseases of the Genitourinary System**	124	35	-72%*
**Diseases of the Digestive System**	80	22	-72%*
**Endocrine, Nutritional and Metabolic Disease and Immunity Disorders**	39	14	-64%*
**Diseases of the Circulatory System**	116	45	-61%*
**Supplementary Classification of Factors Influencing Health Status and Contact with Health Services**	67	27	-60%*
**Symptoms, Signs, and Ill-defined Conditions**	369	151	-59%*
**Mental Disorders**	227	101	-56%*
**Diseases of the Nervous System and Sense Organs**	82	37	-55%*
**Injury and Poisoning**	149	68	-54%*
**Diseases of the Respiratory System**	105	50	-52%*
**Diseases of the Musculoskeletal System and Connective Tissue**	100	49	-51%*
**Diseases of the Blood System and Blood-forming Organs**	22	3	-86%
**Supplementary Classification of External Causes of Injury and Poisoning**	22	11	-50%
**Infectious and Parasitic Disease**	14	8	-43%
**Disease of the Skin and Subcutaneous Tissue**	9	6	-33%
